# Postoperative opioid-induced respiratory depression monitoring in the context of supplemental oxygen: current limitations and emerging machine-learning approaches

**DOI:** 10.3389/fmed.2026.1961833

**Published:** 2026-10-30

**Authors:** Jing Wen, Qiang Fu

**Affiliations:** Department of Anesthesiology, The Third People's Hospital of Chengdu, Chengdu, Sichuan, China

**Keywords:** artificial intelligence, capnography, machine learning, multimodal monitoring, opioid-induced respiratory depression, postoperative respiratory monitoring, pulse oximetry, supplemental oxygen

## Abstract

Opioid-induced respiratory depression (OIRD) may develop after extubation and remain unrecognized during intermittent postoperative observation. Supplemental oxygen is commonly used to prevent or treat postoperative hypoxemia, but it may preserve peripheral oxygen saturation despite impaired ventilation and thereby delay oximetric warning. Experimental evidence suggests that hyperoxia may also reduce peripheral chemoreceptor drive, although the clinical importance of this effect during routine postoperative low-flow oxygen therapy remains uncertain. This narrative Mini Review examines how supplemental oxygen affects the detection and interpretation of postoperative OIRD, evaluates current respiratory monitoring and machine-learning evidence, and considers requirements for clinical translation. Continuous oximetry and capnography detect more respiratory abnormalities than intermittent assessment, but monitor-defined episodes do not necessarily represent clinically important or opioid-attributable respiratory depression. Their interpretation requires consideration of technical signal validity, clinical significance, and opioid contribution. Existing machine-learning studies have predicted threshold-defined respiratory events, classified individual breaths, distinguished normal from abnormal monitoring segments, or quantified ataxic breathing, supporting the technical feasibility of automated signal interpretation and respiratory phenotype discovery. Among the studies reviewed, none used a prospectively adjudicated clinical OIRD outcome or explicitly modeled oxygen delivery as a time-varying exposure. A testable development pathway would integrate respiratory time series with oxygen delivery, medication administration, and changing clinical context, followed by external validation across devices, institutions, and healthcare settings. Clinical value should ultimately be judged by earlier appropriate intervention and reduced respiratory harm without an unacceptable alarm burden, rather than by improved prediction of threshold-defined episodes alone.

## 1 Introduction

Opioids remain widely used within multimodal perioperative analgesia, particularly for moderate-to-severe postoperative pain. Non-opioid analgesics and local-anesthetic-based regional techniques reduce systemic opioid requirements but may not fully replace opioids across all patients and procedures. Neuraxial opioids constitute another opioid exposure with a drug-dependent time course, including delayed respiratory depression after hydrophilic morphine (1). Systemic and neuraxial opioids can produce bradypnea, reduced tidal volume, apnea, or irregular breathing, often accompanied by increasing sedation (2, 3). Reported OIRD incidence depends strongly on ascertainment. Estimates based on naloxone administration range from 0.04% to 0.5%, whereas definitions based on bradypnea or hypoxemia yield 23% to 41% (4). These values describe different event categories rather than conflicting estimates of a single outcome.

During general anesthesia, ventilation is continuously monitored and, when necessary, supported or controlled. Respiratory depression may become apparent after extubation in the postanesthesia care unit (PACU), where early events can signal greater risk after discharge (3). The surveillance gap becomes more consequential on general wards, where observation is intermittent and opioid effects may interact with residual anesthetics, sedatives, sleep, and pre-existing sleep-disordered breathing. In the prospective multicenter PRODIGY trial, blinded continuous oximetry and capnography detected at least one prespecified respiratory depression episode in 46% of 1,335 patients receiving parenteral opioids on general care floors (5). This figure represents monitor-defined abnormalities rather than clinically severe OIRD.

Supplemental oxygen changes how these abnormalities are detected. In Fu et al., a 50% reduction in minute ventilation decreased SpO₂ only in anesthetized surgical patients breathing room air, not at FiO₂ 0.25 or 0.30; in a randomized PACU cohort, desaturation occurred in 9.0% on room air versus 2.3% with oxygen (*p* = 0.02) (6). This demonstrates oxygen-mediated masking of hypoventilation but does not establish potentiation of opioid effects. In a 202-patient PRODIGY analysis, apnea and bradypnea were more frequent during oxygen than room-air exposure, whereas desaturation did not differ (7); because oxygen was not randomized, confounding by indication remains possible. The clinical question is therefore how to assess ventilation when oxygenation changes may lag behind ventilatory decline, not whether indicated oxygen should be withheld. This Mini Review examines postoperative OIRD monitoring in the context of supplemental oxygen, including physiology, monitoring limitations, and machine-learning approaches. Figure 1 is an evidence-to-translation map: solid pathways denote established measurement relationships or observed clinical associations, dashed pathways mainly experimental or mechanistic evidence, and teal pathways a proposal requiring prospective validation.

**Figure 1:**
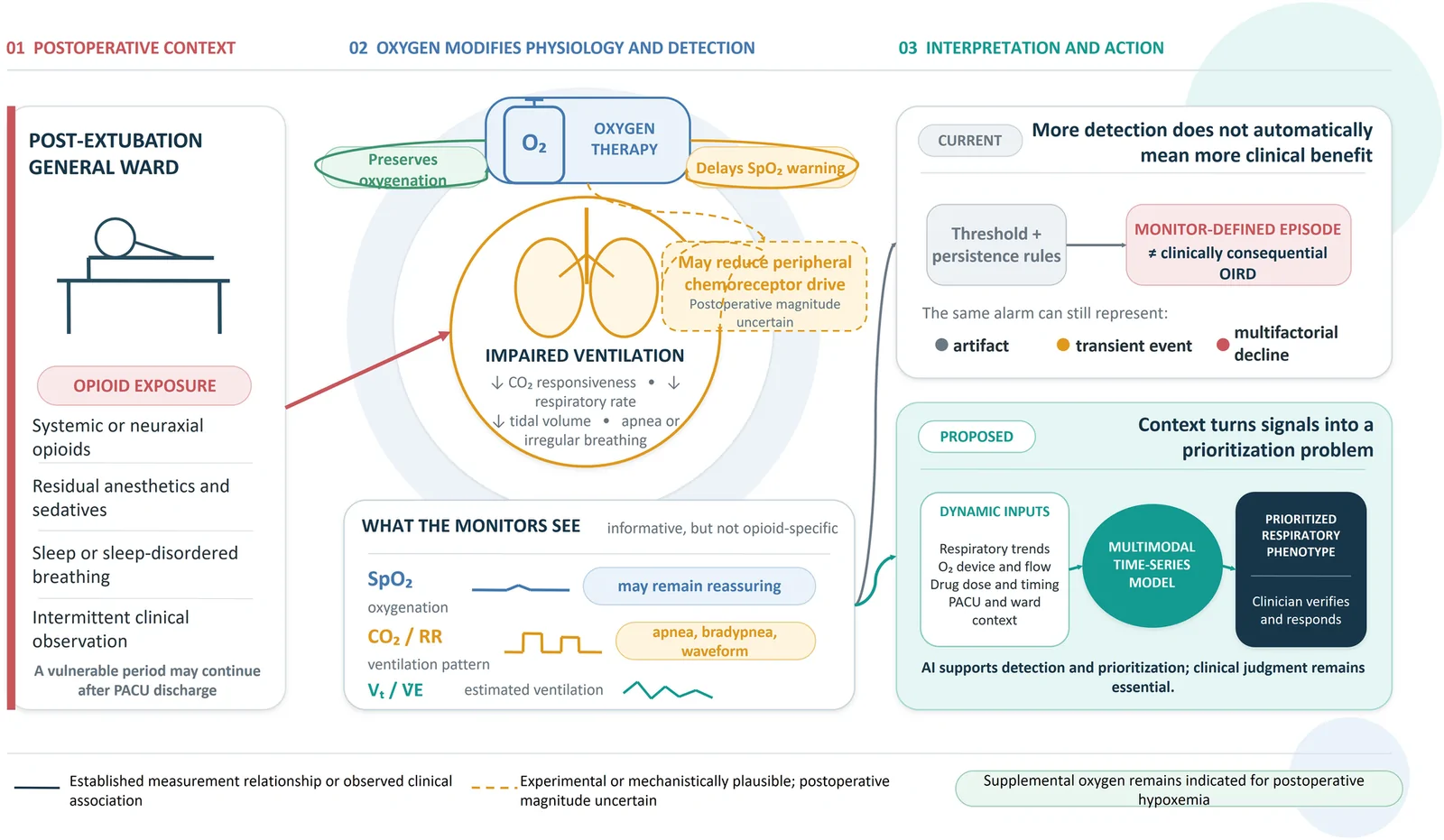
Evidence-to-translation framework for postoperative respiratory monitoring during supplemental oxygen therapy. Supplemental oxygen treats hypoxemia but may preserve SpO₂ during impaired ventilation and delay oximetric warning. Experimental evidence also suggests a possible reduction in peripheral chemoreceptor drive, although its magnitude during routine postoperative therapy remains uncertain. Current threshold-based systems identify respiratory abnormalities without necessarily establishing signal validity, clinical importance, or opioid contribution. The proposed multimodal approach integrates respiratory signals with oxygen delivery, medication administration, and changing clinical context to support clinician assessment; it represents a testable research direction rather than an established standard of care. Solid pathways denote established measurement relationships or observed clinical associations, dashed pathways denote experimental or mechanistically plausible relationships with uncertain postoperative magnitude, and teal arrows denote the proposed pathway requiring prospective validation.

This narrative Mini Review was informed by targeted PubMed searches combining terms for OIRD, supplemental oxygen, postoperative monitoring, capnography, machine learning, and artificial intelligence, with reference-list screening for additional PubMed-indexed studies. The focused evidence map included original human studies applying machine learning to respiratory or oxygenation signals to detect, classify, predict, or phenotype acute opioid-related respiratory abnormalities. We extracted population, inputs, analytical task, reference label, validation, performance, clinical OIRD adjudication, and representation of oxygen therapy. We excluded studies of community overdose, chronic opioid use, genetic susceptibility, or static clinical risk prediction without respiratory-monitoring inputs. The focused evidence-map search was last updated in August 2026, and its principal PubMed terms are provided in the Table 1 note.

**Table 1:** Structured comparison of machine-learning studies included in the focused pubMed evidence map.

Study/source cohort	Population and monitoring inputs	Analytical task and reference label	Validation and main performance	Prospectively adjudicated clinical OIRD outcome	Oxygen delivery as a time-varying input	Principal interpretive gap
**Jungquist et al. (2019)** (17); independent PACU pilot	48 postsurgical PACU patients; pulse oximetry, EtCO₂, minute ventilation, and an opioid-dose indicator	SVM prediction of an author-defined, threshold-based OIRD event up to 10 min before threshold crossing	Performance evaluated within the pilot cohort; combined inputs achieved approximately 80% accuracy at 10 min	**No**; the outcome was defined by prespecified physiological thresholds	**No**; supplemental oxygen was administered, but device, flow, and timing were not modeled dynamically	Small single-center pilot without independent or prospective clinical validation
**Ermer et al. (2020)** (20); NCT02744599	26 healthy volunteers receiving target-controlled propofol and remifentanil; respiratory inductance plethysmography and intranasal pressure	Multiclass SVM quantification of ataxic-breathing severity against ratings from three domain experts	Within-cohort training and testing; Vanbelle *κ* 0.98 for plethysmography and 0.96 for intranasal pressure	**No**; the reference consisted of expert phenotype ratings	**No**	Controlled volunteer study using an expert phenotype reference rather than a postoperative clinical outcome
**Conway et al. (2023)** (18); PRODIGY secondary analysis	400 patients from nine North American sites; 10,391 15-s capnography segments	Convolutional neural-network classification of breath versus no breath against expert waveform labels	Leave-one-hospital-out internal-external validation; accuracy 0.97, precision 0.97, and recall 0.96	**No**; the reference was a breath-level waveform label	**No**	Breath detection is not equivalent to clinically important or opioid-attributable respiratory depression; alarm and outcome effects were not tested
**Spijkerboer et al. (2024)** (19); PRODIGY secondary analysis	1,458 ward patients from 16 sites; 10,145 initially selected 90-s capnography and oximetry segments, of which 2,287 (22.5%) were excluded during preprocessing, leaving 7,858 for modeling	Five ML methods; XGBoost classified normal versus abnormal segments. The abnormal class combined apnea, other respiratory depression, and artifact	Subject-level 80:20 split with stratified fivefold cross-validation in the training set; F₂ 0.94, recall 0.98, and precision 0.83	**No**; expert waveform labels did not establish clinical importance or opioid contribution	**No**; SpO₂ was included, but oxygen device, flow, and timing were not	Artifact was included in the abnormal class; 22.5% of initially selected segments were excluded during preprocessing, which may limit generalizability to continuous monitoring with missing or poor-quality signals; no region-held-out or prospective clinical validation
**Farney et al. (2025)** (21); NCT02744599 secondary analysis	The same 26-volunteer remifentanil/propofol cohort; respiratory inductance plethysmography, respiratory rate, and alertness assessment	SVM-derived ataxic-breathing severity compared with conventional OIRD defined by bradypnea and reduced alertness	121 steady-state assessments; sensitivity 92% and specificity 28% for at least moderate ataxic breathing	**No**; the reference was a conventional physiological and alertness threshold	**No**	Low specificity and use of the same volunteer cohort as Ermer et al.; postoperative specificity and clinical significance remain unknown

Performance metrics should not be compared directly across rows because the studies differed in populations, inputs, analytical tasks, reference labels, and validation strategies. Prospectively adjudicated clinical OIRD was defined as an outcome incorporating physiological deterioration, opioid contribution, and need for clinical intervention; expert waveform or phenotype labels and threshold-based definitions alone were not counted. Time-varying oxygen input was defined as oxygen device, prescribed flow, and timing; SpO₂ alone was not counted. Conway et al. and Spijkerboer et al. analyzed PRODIGY data, whereas Ermer et al. and Farney et al. analyzed NCT02744599. **Search strategy: The** focused PubMed evidence map used two Title/Abstract searches. The first combined (“opioid-induced respiratory depression” OR “opioid-induced ventilatory impairment” OR OIRD OR OIVI) with (“machine learning” OR “deep learning” OR “artificial intelligence” OR “neural network” OR “support vector machine” OR XGBoost OR classifier OR “predictive model”). The second combined (opioid* OR remifentanil) with (capnograph* OR oximetr* OR “minute ventilation” OR plethysmograph* OR “respiratory waveform” OR “ataxic breathing”) and (“machine learning” OR “deep learning” OR “neural network” OR “support vector machine” OR classifier* OR algorithm*). Searches were last updated in August 2026.

## 2 Supplemental oxygen in OIRD: detection, ventilatory control, and exposure variability

SpO₂ reflects hemoglobin saturation, not alveolar ventilation or CO₂ clearance. Supplemental oxygen may therefore preserve SpO₂ as minute ventilation falls and PaCO₂ rises, delaying rather than preventing oximetric detection of hypoventilation. This limitation of oxygenation-based detection does not imply that clinically indicated oxygen should be withheld.

Signal masking should be distinguished from the less established possibility that hyperoxia alters ventilatory control. Opioids reduce central CO₂ responsiveness, whereas peripheral chemoreceptors may provide compensatory drive. In healthy volunteers, the same remifentanil bolus produced greater reductions in minute ventilation and more apnea during hyperoxia at an FiO₂ of 0.5 than during normoxia, while attenuating the SpO₂ fall (8). Spiller et al. reviewed preclinical evidence that peripheral chemoreflexes may protect against OIRD (9); in an original rat study, bilateral carotid sinus nerve transection exacerbated morphine-induced respiratory depression (10). These studies establish biological plausibility but not whether routine postoperative low-flow oxygen materially deepens OIRD. Delayed oximetric warning is therefore better supported than clinically meaningful physiological potentiation.

Low-flow nasal cannula oxygen is not a fixed exposure. Direct tracheal and pharyngeal sampling shows that delivered FiO₂ varies within and between individuals, rising with oxygen flow and falling as respiratory rate and minute ventilation increase; breathing route and pattern also contribute (11, 12). Device, flow, and timing are therefore proxies rather than direct exposure measurements. Because respiratory pattern changes during OIRD, a static oxygen present-or-absent variable loses relevant information. Monitoring and analytical models should assess ventilation independently of oxygenation and treat oxygen delivery as time-varying clinical context. This variability also limits comparisons of oxygen-treated and room-air periods without detailed temporal data.

## 3 Respiratory monitoring and the limits of OIRD identification

Once oxygenation and ventilation may diverge, monitor performance cannot be judged only by whether a device crosses a threshold. Current monitors detect abnormalities relevant to OIRD but cannot establish opioid causation or clinical importance. Pulse oximetry measures oxygenation, capnography provides respiratory rate, apnea duration, waveform, and end-tidal carbon dioxide information, and respiratory volume monitoring estimates tidal and minute ventilation. Combining oxygenation and ventilation signals can reveal discordant patterns during supplemental oxygen, but no signal alone determines whether an event is technically valid, clinically important, or primarily caused by opioids.

Adding capnography addresses part of the oxygenation-ventilation mismatch, but it introduces a different acquisition problem. In spontaneously breathing patients, oxygen delivery can dilute nasal carbon dioxide samples or degrade the capnogram when oxygen delivery and carbon dioxide sampling are not adequately separated. Comparative studies show that interference depends on cannula design, with physically separated delivery and sampling pathways producing more reliable end-tidal carbon dioxide measurements than shared-pathway designs (13, 14). Supplemental oxygen does not uniformly invalidate capnography, but it makes signal reliability partly device-dependent.

For the purposes of this review, interpretation can be organized along three dimensions. Technical validity asks whether the apparent event reflects respiration or artifact caused by movement, cannula displacement, mouth breathing, or sampling dilution. Clinical significance distinguishes a brief, self-limited abnormality from sustained, recurrent, or progressively worsening respiratory compromise. Opioid attribution asks whether opioids were the principal cause, a contributing factor, or incidental to an event driven mainly by residual anesthesia, other sedatives, sleep-disordered breathing, airway obstruction, or pulmonary disease (15). Respiratory support should follow physiological urgency without awaiting opioid attribution.

Threshold-based definitions do not resolve these dimensions, even when persistence criteria are included. A threshold-defined episode may be technically valid but transient, or clinically important but predominantly non-opioid in origin. Conversely, supplemental oxygen may prevent an SpO_2_ threshold from being crossed despite worsening ventilation. Binary event labels can therefore combine different respiratory phenotypes while missing others. This creates a target-definition problem for machine learning: better prediction of a monitor-defined label is not equivalent to better recognition of clinically important respiratory deterioration to which opioids materially contributed. The distinction matters because a model can only learn the outcome represented by its training labels. Table 1 provides a structured comparison of the PubMed-indexed machine-learning studies included in this review, including their populations, monitoring inputs, analytical targets, reference labels, validation strategies, and principal interpretive gaps.

## 4 Machine learning for postoperative respiratory monitoring: current evidence and transferability

Outside the focused evidence map, baseline tools estimate susceptibility, whereas the included studies address prediction or real-time interpretation of respiratory signals; these tasks require different inputs, labels, and validation. The PRODIGY score estimates baseline risk from five fixed variables (5), whereas STOP-Bang screens for obstructive sleep apnea rather than OIRD (16). Both can guide initial monitoring but do not update with postoperative opioids, sedatives, oxygen delivery, PACU events, or evolving respiratory status.

In a single-center PACU pilot, Jungquist et al. monitored 48 postsurgical patients using pulse oximetry, capnography, respiratory volume monitoring, and opioid administration (17). A support vector machine combining these inputs predicted threshold-defined events 10 min before threshold crossing with approximately 80% accuracy. Supplemental oxygen was administered, but its device, flow, and timing were not modeled dynamically. Limitations included the small cohort, an author-defined physiological outcome, and no independent validation.

Conway et al. trained a convolutional neural network on 10,391 15-second capnography segments from 400 patients at nine North American PRODIGY sites. Breath-versus-no-breath classification achieved 0.97 accuracy, 0.97 precision, and 0.96 recall using leave-one-hospital-out validation (18). This demonstrated breath-level transport across participating hospitals but did not evaluate false alarms, clinically adjudicated OIRD, or patient outcomes.

Spijkerboer et al. evaluated combined capnography and oximetry from PRODIGY sites in Asia, Europe, and the United States. XGBoost distinguished expert-labeled abnormal from normal 90-second segments with an F_2_ score (β = 2) of 0.94, recall of 0.98, and precision of 0.83 (19). Because the abnormal class combined apnea, other respiratory depression, and artifact, the model did not separate artifact from physiological compromise or determine opioid contribution.

Ermer et al. developed a multiclass support vector machine to quantify ataxic-breathing severity in 26 healthy volunteers receiving propofol and remifentanil (20). Using respiratory inductance plethysmography or intranasal pressure signals, agreement with three experts yielded Vanbelle *κ* values of 0.98 and 0.96, respectively. However, the reference standard was an expert-rated phenotype rather than clinically adjudicated OIRD, and oxygen delivery was not modeled dynamically.

Using the same volunteer cohort and SVM-derived score, Farney et al. evaluated ataxic breathing against a conventional OIRD definition based on bradypnea and reduced alertness (21). Moderate or greater ataxic breathing had 92% sensitivity and 28% specificity and occurred in 72% of conventionally negative assessments. This represents phenotype discovery rather than postoperative detector validation; its clinical significance and specificity in postoperative patients remain unknown.

Table 1 shows that these five publications, derived from three principal data sources, addressed noncomparable intermediate tasks. None used a prospectively adjudicated clinical OIRD outcome incorporating physiological deterioration, opioid contribution, and need for intervention, or represented oxygen delivery as a time-varying input. Their technical performance therefore does not establish bedside OIRD detection.

Collectively, these studies represent development rather than deployment. Jungquist et al. evaluated a small PACU cohort, Ermer et al. and Farney et al. analyzed the same volunteer cohort, and Conway et al. and Spijkerboer et al. performed secondary PRODIGY analyses. PRODIGY included three continents, but regional opioid exposure differed substantially (22). Conway et al. withheld hospitals within North America, whereas Spijkerboer et al. used a patient-level random split; the PACU and volunteer studies lacked external validation. Performance may also vary with oxygen protocols, cannula and monitor design, patient characteristics, and alarm-response workflows. Evidence therefore supports the technical feasibility of automated signal interpretation and respiratory phenotyping, but not identification of clinically adjudicated postoperative OIRD or improved outcomes.

## 5 Discussion

Supplemental oxygen remains clinically indicated for postoperative hypoxemia, but its administration changes how respiratory monitoring should be interpreted. The evidence for delayed oximetric warning during impaired ventilation is stronger than the evidence that routine postoperative oxygen materially potentiates OIRD. The practical implication is not a different threshold for giving oxygen, but a different expectation of what SpO_2_ alone can reveal once oxygen has been administered. This distinction should guide both clinical interpretation and future model development.

The observational association reported by Doufas et al. warrants quantitative qualification. Of 258 patients enrolled at two United States PRODIGY sites, 56 (21.7%) were excluded because of incomplete oxygen records, leaving 74 patients who remained on room air, 88 who received oxygen intermittently, and 40 who received oxygen continuously (7). The proportions experiencing at least one monitor-defined OIRD episode were 54.1%, 60.2%, and 62.5%, respectively, and did not differ significantly (*p* = 0.615). However, after accounting for exposure time and within-patient correlation using generalized estimating equations, supplemental-oxygen exposure was associated with higher rates of all OIRD episodes (IRR 2.7, 95% CI 1.4–5.1), apnea (IRR 2.8, 95% CI 1.5–5.2), and bradypnea (IRR 3.0, 95% CI 1.2–7.9). A within-patient analysis of the intermittent-oxygen group showed a similar direction of association. These analyses address recurrent monitor-defined event rates rather than the probability of clinically important deterioration. Moreover, oxygen administration was neither randomized nor protocolized, and initiation or discontinuation may have followed hypoxemia, sedation, or early respiratory decline, producing time-varying confounding by indication. The two-site design, exclusion of patients with incomplete oxygen records, indirect characterization of inspired oxygen exposure, and absence of patient-centered outcomes limit causal attribution but do not diminish the study's value in identifying a clinically relevant association and motivating prospective evaluation.

Whether this interpretive change improves care depends on what happens after an abnormal signal is detected. The case for enhanced respiratory surveillance is strongest at the level of event detection and less conclusive at the level of patient outcomes. In a before-and-after concurrence study, Taenzer et al. reported reductions in rescue events and intensive care unit transfers after continuous pulse oximetry was linked to direct wireless notification of nursing staff (23). A subsequent systematic review found that continuous oximetry increased recognition of desaturation and that capnography detected respiratory depression more frequently than pulse oximetry alone (24). However, randomized evidence included in that review showed no reduction in intensive care unit transfer or mortality, the pooled reduction in intensive care transfer did not reach statistical significance, and no capnography study evaluated mortality or intensive care transfer. A later review similarly found that oxygen desaturation was the only outcome consistently detected more effectively by continuous pulse oximetry in ward patients (25).

The mixed outcome evidence may therefore reflect differences in the intervention being tested rather than a simple opposition between monitoring and no monitoring. A device identifies abnormalities. A surveillance system must also suppress or prioritize nonactionable alarms, deliver notifications to an available clinician, and support an appropriate response. Taenzer et al. evaluated such an integrated notification system, but the observational design cannot exclude concurrent changes in care. Conversely, neutral outcome findings from device-centered studies do not show that earlier recognition lacks clinical value. They may reflect transient event labels, ineffective notification pathways, delayed responses, or limited statistical power for rare severe outcomes. Existing evidence supports improved detection more clearly than it identifies which combination of signals, alarm logic, staffing, and response protocols prevents serious harm.

Whether surveillance should be universal or risk-stratified also remains unresolved. Universal monitoring reduces dependence on imperfect baseline screening and may detect deterioration in patients not initially considered high risk. Its disadvantages include equipment and staffing requirements, patient discomfort, signal loss, and alarm burden. Risk-stratified monitoring is more feasible where resources are limited, but baseline scores do not reflect subsequent opioid doses, sedative co-administration, oxygen adjustments, PACU events, or changing respiratory patterns. A hybrid approach could use baseline risk to determine initial monitoring intensity and then update surveillance when postoperative exposures or respiratory status change. This approach is clinically plausible but requires prospective validation.

The evidence base has several limitations. OIRD definitions range from naloxone administration or escalation of care to monitor-defined bradypnea, apnea, desaturation, or carbon dioxide abnormalities, limiting comparison across studies. The association between supplemental oxygen and apnea or bradypnea in postoperative patients is observational and susceptible to confounding by indication. Evidence for physiological potentiation comes mainly from preclinical work and controlled volunteer studies. Machine-learning evidence is dominated by secondary analyses of PRODIGY data and small experimental cohorts, with limited independent validation. The focused PubMed evidence map was restricted to predefined acute opioid-related monitoring studies and did not include formal risk-of-bias assessment or meta-analysis; it should not be interpreted as exhaustive of all OIRD or early-warning literature. Its focus on adult postoperative ward care also limits extrapolation to pediatric patients, critical care, procedural sedation, and community opioid exposure.

Relevant benchmarks already exist outside opioid-specific respiratory monitoring. Churpek et al. developed eCARTv5 using demographics, vital signs and their trends, clinical documentation, and laboratory values, followed by retrospective and prospective validation across 21 hospitals (26). TREWS further demonstrated that deployment cannot be evaluated separately from clinician interaction and the subsequent response pathway: among patients identified before antibiotic treatment, provider confirmation of an alert within 3 h was associated with improved outcomes, although the observational design did not establish causality (27). Conversely, external validation of the widely implemented Epic Sepsis Model found an AUROC of 0.63 and substantial alert burden, with alerts generated for 18% of admissions while 67% of sepsis cases were missed (28). These systems do not resolve the OIRD-specific problems of opioid attribution, oxygen-mediated signal masking, or respiratory-waveform interpretation. They do, however, establish relevant benchmarks for external validation, calibration, alert burden, workflow integration, and outcome evaluation. The contribution proposed here is therefore the adaptation of established early-warning-system principles to postoperative OIRD, rather than a new generic early-warning architecture.

Building on these benchmarks, an OIRD-specific development pathway should evaluate an integrated monitoring-and-response system rather than predictive performance alone. Oxygen-aware models could represent oxygen-delivery device, prescribed flow, and timing as time-varying clinical proxies, while recognizing that these variables do not directly measure delivered FiO₂. These inputs could be combined with respiratory waveforms, opioid dose and route, other sedatives, baseline susceptibility, and preceding PACU events. Model outputs should distinguish technical signal validity, physiological severity, and probable opioid contribution rather than collapse these dimensions into a single binary OIRD label. In practice, technical validity could be evaluated against expert-adjudicated artifact labels, physiological severity against event duration, recurrence, multichannel concordance, and need for intervention, and opioid contribution against temporally aligned opioid and sedative exposure together with adjudicated competing causes. Following internal development, independent validation across hospitals, devices, patient populations, and healthcare systems should assess discrimination, calibration, missingness, and subgroup performance. Silent prospective evaluation should then determine lead time and potential alert burden before model outputs are shown to clinicians. Pragmatic or cluster-randomized trials could subsequently evaluate nonactionable alerts, time to clinical assessment, respiratory support, opioid reversal, escalation of care, and clinically important respiratory events. The 2026 international consensus conference identified artificial intelligence and wearable monitoring as OIRD research priorities (29), but the value of an oxygen-aware approach remains to be demonstrated. Success should ultimately be defined by earlier appropriate intervention and reduced harm without an unacceptable alarm burden, rather than by improved prediction of threshold-defined episodes alone.
